# Innovating Dementia Support: An Evaluation of Cost‐Effectiveness and Wellbeing Outcomes of the Sage House Model

**DOI:** 10.1002/alz70858_102222

**Published:** 2025-12-25

**Authors:** Rachel Lousie King, Stuart Warren, Elizabeth Vass, Kian Beaumont, Stewart Seymour, Samuel Bell, Benjamin Sharpe, Rosana Pacella, Antonina Pereira

**Affiliations:** ^1^ University of Chichester, Chichester, United Kingdom; ^2^ Sussex Partnership NHS Foundation Trust, Dementia Research Unit, Crowborough, United Kingdom; ^3^ Public Health and Social Research Unit, Chichester, United Kingdom; ^4^ Elysium Heathcare, Hove, United Kingdom; ^5^ University of Greenwich, London, United Kingdom

## Abstract

**Background:**

Today, nearly one million people live with dementia (PLWD) in the UK, a number projected to rise to 1.7 million with estimated costs of £90 billion by 2040. ^1–4^ These projections highlight the necessity to develop cost‐effective solutions to providing care.

Multicomponent supportive care approaches (MSCA) integrate tailored support and psychosocial interventions to enable a personalised support package, ^5^ showing promise in enhancing wellbeing for PLWD and care partners, while offering cost‐effective care solutions. ^5, 6^ However, these approaches are underutilised due to the additional implementation complexities inherent with multifaceted intervention strategies.

The Sage House Model is a MSCA that has overcome these challenges by utilising a collaborative approach between the voluntary and healthcare sectors, integrating a range of specialised dementia services into an accessible community‐based centre. The present study aimed to investigate the wellbeing and economic impact of the Sage House Model of dementia support.

**Method:**

A natural experiment was run comparing wellbeing (QoL, Wellbeing, Life Satisfaction) and economic outcomes (Health and Social Care Engagement) between a group of participants with access to the Sage House Model and a group receiving standard care. The sample included 132 PLWD (M_age_ 74.64, SD 8.30) and 129 care partners (M_age_ 67.23, SD 9.84).

**Result:**

It was observed that PLWD with access to the Sage House Model reported higher QoL (*p* = .004, ω^2^ = .06), wellbeing (*p* = .044, ω^2^ = .03) and life satisfaction (*p* = .004, ω^2^ = .07) as compared to the group receiving standard care. Care partners with access also reported greater needs‐based QoL (*p* = .005, ω^2^ = .07) relating to improved access to support and information. It was also observed that participants with access to the Sage House Model cost health and social care less over a three‐month period (*p* = .038, ω^2^ = .02) and had greater Health Related QoL (*p* = .004, ω^2^ = .03). After incorporating costs associated with funding access, the model continued to demonstrate cost‐effectiveness.

**Conclusion:**

Overall, this study provides initial evidence that suggests that the Sage House Model offers a scalable, community‐driven approach to improving dementia care outcomes and supporting PLWD and care partners, while reducing economic strain on health systems.

